# Cardiac DPD-uptake time dependency in ATTR patients verified by quantitative SPECT/CT and semiquantitative planar parameters

**DOI:** 10.1007/s12350-022-03149-4

**Published:** 2022-12-13

**Authors:** Tim Wollenweber, Rene Rettl, Elisabeth Kretschmer-Chott, Sazan Rasul, Oana Cristina Kulterer, Kilian Kluge, Franz Duca, Diana Bonderman, Marcus Hacker, Tatjana Traub-Weidinger

**Affiliations:** 1 Division of Nuclear Medicine, Department of Biomedical Imaging and Image-Guided Therapy, Medical University of Vienna, Vienna, Austria; 2 Division of Cardiology, Department of Internal Medicine II, Medical University of Vienna, Vienna, Austria; 3 5th Medical Department with Cardiology, Clinic Favoriten, Vienna, Austria

**Keywords:** Amyloidosis, quantification, ATTR, SPECT/CT, SUV, therapy monitoring

## Abstract

**Background:**

Bone scintigraphy plays an important role in the diagnosis of cardiac Transthyretin-Related Amyloidosis (ATTR). The mechanism of myocardial tracer accumulation and its dependence over time are not fully understood. Recently, a scintigraphic quantification of the cardiac amyloid deposition has been discussed. Nevertheless, little is known regarding the right time of quantitative imaging.

**Methods:**

The geometrical mean of decay corrected total counts over the heart and the heart/whole-body ratio (H/WB) were evaluated in 23 patients undergoing DPD-bone scan with planar whole-body images 1 and 3 hours post injection (p.i.). Myocardial standard uptake values (SUV)peak were assessed in another 15 patients with quantitative SPECT/CT imaging 1 hours and 3 hours p.i..

**Results:**

Total counts over the heart (1 hours p.i.: 81,676 cts, range 69,887 to 93,091 cts and 3 hours p.i.: 64,819 cts, range 52,048 to 86,123 cts, *P* = .0005) and H/WB ratio (1 hours p.i.:0.076 ± 0.020 and 3 hours p.i. 0.070 ± 0.022; *P* = .0003) were significantly increased 1 hours p.i.. Furthermore median myocardial SUVpeak (1 hours p.i.:12.2, range 9.6 to 18.9 and 3 hours p.i.: 9.6, range 8.2 to 15.0, *P* = 0.0012) was also significantly higher after 1 hours p.i. compared to 3 hours p.i..

**Conclusion:**

Cardiac DPD activity and myocardial SUVpeak are time-dependent, which should be considered when using quantitative bone scintigraphy in ATTR patients.

**Supplementary Information:**

The online version contains supplementary material available at 10.1007/s12350-022-03149-4.

## Introduction

Amyloidosis is a group of rare diseases originated by deposition of misfolded proteins leading to progressive multiorgan failure and death.^1^ The different types of amyloidosis include light-chain amyloidosis (AL), amyloid amyloidosis (AA), wild-type transthyretin amyloidosis (wtATTR) and the hereditary transthyretin amyloidosis (hATTR).^2^ ATTR is caused by instability of the tetramere transthyretin, a transport protein of thyroxine and retinol A with consecutive formation of misfolded aggregates from monomeres as insoluble amyloid fibrils with deposit in various organs. Typically, the heart, kidneys, peripheral nervous system and liver are affected. Whereas hATTR is related to mutations leading to destabilization of the TTR protein, wtATTR has an inherent, albeit low potential to form amyloid fibrils.^3,4^

Imaging studies have shown that radiolabeled phosphonates, such as [^99m^Tc]-3,3-diphosphono-1,2-propanodicarboxylic acid (DPD), have a strong affinity for TTR amyloid fibrils in the heart. The exact mechanism of binding is not fully understood, but a high density of microcalcifications of ATTR seems to play an important role.^5,6^

Recently, several drugs have been developed for ATTR which have shown to reduce mortality in patients with known cardiac ATTR (cATTR).^7,8^ Therefore, the repetitive quantification of amyloid burden using imaging modalities for treatment monitoring has become increasingly important. Several studies have demonstrated the feasibility of standard uptake values (SUV) quantification of amyloid deposition on bone scintigraphy using SPECT/CT.^9–15^ However, quantification of cardiac amyloid deposition is subject to several drawbacks. There is a competition for the tracer uptake in the different compartments such as heart, soft tissue and bone.^15^ To correct for this influence on the SUV quantification, Scully and co-workers have developed a SUV retention index as recently described.^12^ In addition, cardiac tracer uptake does not appear to be constant over time.^16,17^ Therefore, the aim of this study was to track the time course of myocardial DPD tracer accumulation in patients with ATTR amyloidosis using planar and quantitative SPECT/CT imaging for better understanding of the tracer behavior and establishing a potential imaging protocol for therapy assessment.

## Methods

### Study population

Thirty-eight consecutive patients with verified ATTR amyloidosis [10 women and 28 men; age 79 ± 8 years, 33 patients wtATTR, 5 patients with hATTR (1 × Val50Met, 4 × His108Arg)] were considered for this study on the basis of the scintigraphic imaging protocols performed for cardiac amyloidosis evaluation.

Heart failure stage NYHA 1 was known in 4 patients, stage NYHA 2 in 13 patients and stage NYHA3 in 1 of the 38 patients. Heart failure with preserved ejection fraction (HFpEF) was observed in 14 patients and heart failure with reduced ejection fraction (HFrEF) 9 of 38 patients. Three patients had an additional diagnosis of diabetes mellitus type II (DM II) and 9 patients a diagnosis of coronary heart disease (CHD). Furthermore, polyneuropathy was documented in 12 patients and arterial hypertension in 26 of 38 patients. All patients showed elevated NT-ProBNP levels above 150 pg/mL with above 1500 pg/mL seen in 28 patients and below 1500 pg/mL in 10 patients (mean ProBNP levels: 3197 ± 5933 pg/mL).

Cardiac MRI (cMRI) was performed in 22 patients and echocardiography in 35 patients, while 19 of the 38 patients had both cMRI and echocardiography.

On echocardiography left ventricular wall thickening greater than 12 mm was seen in all patients (mean septal thickness IVS 19.0 ± 5.0 mm and mean posterior wall thickness PWT 14.7 ± 3.2 mm) with additional findings such as Grade 2 or worse diastolic dysfunction, a reduced tissue Doppler s0, e0, and a0 waves velocities (< 5 cm/s) or a multiparametric echocardiographic score > 8 points as described by Garcia-Pavia.^18^

In MRI all 23 imaged patients showed the full picture of cardiac amyloidosis with diffuse subendocardial late gadolium enhancement (LGE) in 15 patients or partly diffuse transmural LGE the remaining 8 patients. An abnormal myocardial nulling was observed in 9 patients. The extracellular volume (ECV) was > 40% in 23 patients and less than 40% in 3 patients (mean ECV 50 ± 14.0%). Moreover, MRI revealed left ventricular LVEF below 55% in 16 patients and above 55% in 10 patients with a mean left ventricular ejection fraction on MRI LVEF of 47.7 ± 11.4.

The diagnosis of the disease was made by cardiac MRI (cMRI), echocardiography criteria respectively, Perugini grading (grades 2 and 3) and negative monoclonal protein in 34 patients, while it was made by myocardial biopsy in 3 patients.^18^ One patient with hATTR and the diagnosis of polyneuropathy showed no tracer uptake of the heart with additionally inconspicuous transthoracic echocardiography (TTE). In this patient the diagnosis was genetically verified (His108Arg mutation). For further details of the study population see Table 1. Each individual in this study agreed to the enhanced imaging protocol and gave written informed consent prior to imaging. Appropriate ethical approval was obtained by the Ethics Committee of the Medical University of Vienna (EK #769/2010).

**Table 1:** Patient population

	n	Mean age (years)	Gender (m/w)	Diagnosis finding	Assignment to the groups based on imaging (group1/group2)
wtATTR	33	81 ± 6	26/7	Myocardial biopsy (n = 2); cMRI or echocardiography, Perugini grading and negative monoclonal protein (n = 31)	19/14
hATTR	5	67 ± 7	2/3	Myocardial biopsy (n = 1); cMRI or echocardiography, Perugini grading and negative monoclonal protein (n = 3); polyneuropathy and genetically verified His108Arg mutation (n = 1)	4/1
	1 × Val50Met, 4 × His108Arg				

### Bone scan

Bone scans were performed on a dedicated SPECT/CT system (Symbia Intevo, Siemens Medical Solutions AG, Erlangen, Germany) equipped with a low-energy high-resolution collimator. Planar images were acquired from anterior and posterior with a continuous table feed of 20 to 25 cm per min (ca. 1.000.000 cts) using a 1024 × 256 matrix and a 20% energy window around the 140 keV peak. Tracer activities of 713 ± 15 MBq [Tc^99m^]-DPD were injected intravenously prior to bone scintigraphy SPECT imaging as recently described^15^ was performed using a 180° configuration, 64 views, 20 s per view, 256 × 256 matrix and an energy window of 15% around the 99mTc photopeak of 141 keV. Subsequent to the SPECT acquisition, a low-dose CT scan was acquired for attenuation correction (130 kV, 35 mAs, 256 × 256 matrix, step-and-shoot acquisition with body-contour). SPECT/CT images were reconstructed with the iterative xSPECT/CT QUANT algorithm (eight iterations, four subsets, 3.0 mm smoothing filter, and a 20 mm Gaussian filter) using a 3% National Institute of Standards and Technology (NIST) traceable precision source.

### Image analysis

For analysis of both planar and SPECT/CT images a commercially available software package (Hermes Hybrid 3D software, Hermes Medical Solutions, Stockholm, Sweden) was used. This software package was used to generate region of interests (ROI) in planar images for determining total counts respective mean counts and volumes of interests (VOI) in SPECT/CT images. Planar images performed 1 and 3 hours p.i. were evaluated according to the four-point visual scoring system of Perugini et al.^19^ The images were analyzed by two experienced nuclear physicians. Discrepancies were solved by consensus. For semiquantitative analysis of cardiac uptake in planar images similar to Hutt et al. a region of interest (ROI) was drawn over the heart excluding the sternum in the anterior images.^16^ A similar ROI was drawn over the heart in posterior images. After decay correction the geometric mean of the two was calculated. For further analysis the heart to contralateral (H/CL) ratio was calculated by determining the mean counts per pixel over the heart and the contralateral lung and the heart/whole-body (H/WB) ratio were also assessed as described previously.^20–23^ For SPECT/CT imaging a volume of interest (VOI) was generated using a 39% of the maximal activity threshold for determination of the myocardial standard uptake value (SUV)peak as already earlier described and recommended by Wollenweber et al.^15^ From this VOI the uptake volume of the DPD-uptake was also determined. Moreover, a cubic VOI was placed in the center of an intact vertebral body of the thoracic spine for determination of bone SUVpeak. The SUV retention index was then calculated as described by Scully et al.^12^ Bloodpool activity was measured by placing 1.9 mL VOI in the thoracic aorta at the level of thoracic vertebral body 8.

### Statistical analysis

The MedCalc v19.1 (Ostend, Belgium) software was used for data analysis. The Kolmogorov–Smirnov test was used to check for normal distribution. Normally distributed data were expressed as mean ± standard deviation and non-normally distributed data were expressed as medians and ranges. Comparisons between groups with continuous normally distributed data were compared using the paired students *t* test. If the data were ordinally scaled or not normally distributed the Wilcoxon signed-rank test was used for the comparison between both groups. Ordinally scaled data were presented as mean and standard deviation. Pearson’s correlation was performed to describe the relationship between continuous variables. Obtained *P* values < .05 were considered as statistically significant.

## Results

Based on the imaging procedures 23 patients (7 women, 16 men; age 78 ± 8 years) received planar 2-phase whole-body DPD-bone imaging 1.0 ± 0.4 and 2.9 ± 0.4 hours p.i.. Further 15 patients (3 women, 12 men; age 79 ± 8 years) underwent quantitative 2-phase SPECT/CT 1 hours p.i. (1.0 ± 0.2 hours) and 3 hours p.i. (2.9 ± 0.3).

### Planar image analysis

**Figure 1:**
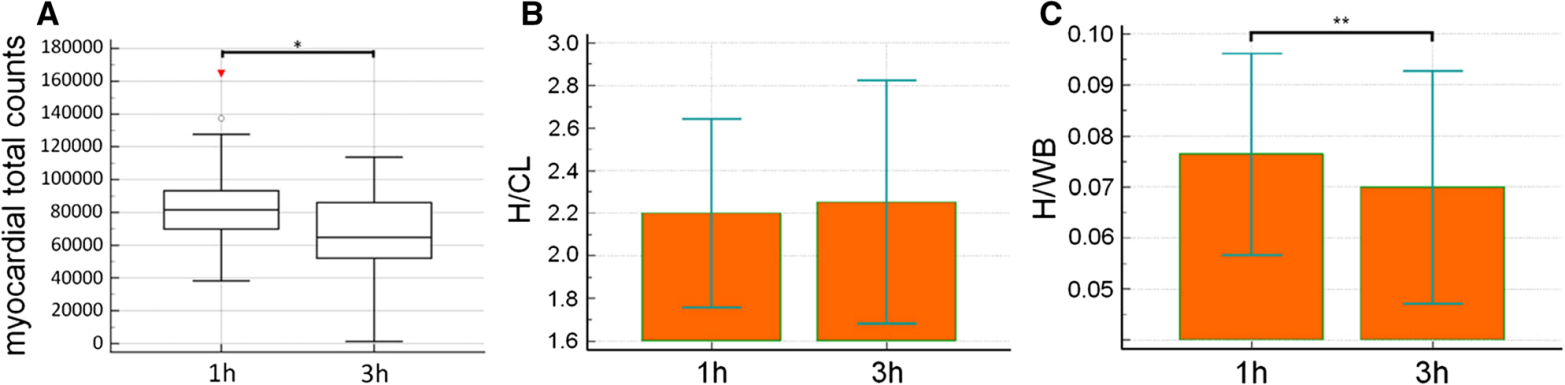
Time-dependent DPD tracer uptake kinetics of the heart using planar imaging. **A** The decay corrected geometric mean of the myocardial total counts was significantly reduced 3 hours p.i. compared to 1 hours p.i. (1 hours p.i: 81,676 cts, range 69,887 to 93,091 cts and 3 hours p.i.: 64,819 cts, range 52,048 to 86,123 cts, *P = .0005). **B** For the H/CL 1 hours and 3 hours p.i., no significant difference could be observed between 1 and 3 hours p.i. (1 hours p.i: 2.2 ± 0.44 and 3 hours p.i 2.25 ± 0.57, *P* = .24). **C** The H/WB was significantly increased 1 hours p.i. compared to 3 hours p.i. (1 hours p.i.: 0.076 ± 0.020 and 3 hours p.i.: 0.070 ± 0.022; **P = .0003)

Perugini grading revealed a significantly higher Perugini score 1 hours p.i. compared to 3 hours p.i. (2.87 ± 0.63 vs 2.48 ± 0.73; *P* = .0039). In 9/23 patients (39%) Perugini score change from 3 to 2, while in 14/23 patients (61%) the Perugini score remained constant over time. The cardiac DPD-uptake based on the decay corrected geometric mean of the total counts over the heart was significantly higher at the earlier time point of measurement (1 hours p.i: 81,676 cts, range 69,887 to 93,091 cts and 3 hours p.i.: 64,819 cts, range 52,048 to 86,123 cts, *P* = .0005**, **Figure 1a). The semiquantitative evaluation of the planar images revealed no significant difference for the H/CL over time (1 hours p.i: 2.2 ± 0.44 and 3 hours p.i 2.25 ± 0.57, *P* = .24, Figure 1b), whereas the H/WB was significantly increased in the early images (1 hours p.i.: 0.076 ± 0.020 and 3 h p.i.: 0.070 ± 0.022; *P* = .0003, Figure 1c).

Furthermore, both patients with HFrEF and with HFpEF showed no significant difference for H/CL between 1 and 3 hours p.i. (HFrEF: H/CL 1 hours p.i.: 2.18 ± 0.56 vs 3 hours p.i.: 2.24 ± 0.76; *P* = .50; HFpEF¸H/CL1h p.i.: 2.36 ± 0.36 vs 3 hours p.i.: 2.34 ± 0.50; *P* = . 78). Even if patients with wtATTR or hATTR were analysed separately, no significant difference was observed (wtATTR H/CL 1 hours p.i.: 2.31 ± 0.44 vs 3 hours p.i.: 2.36 ± 0.57; *P* = .44; hATTR H/CL 1 hours p.i.: 1.91 ± 0.36 vs 3 hours p.i. 1.81 ± 0.38; *P* = .12).

Additionally, linear regression analysis showed a significant correlation for H/WB (*r*^2^ = 0.91; *P* < .0001) and for H/CL between 1 and 3 hours p.i (*r*^2^ = 0.72; *P* < .0001).

### Quantitative SPECT/CT imaging

**Figure 2:**
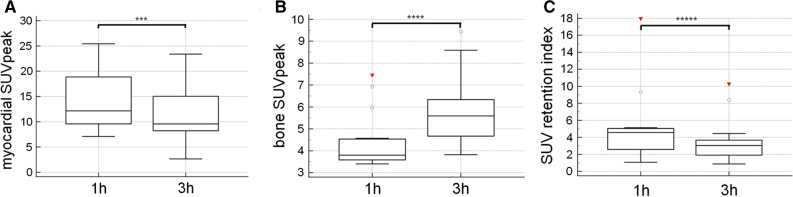
Time-dependent DPD tracer uptake kinetics using quantitative SPECT analysis. **A** Myocardial SUV peak was significantly increased 1 hours p.i. compared to 3 hours p.i. (1 hours p.i.: median SUVpeak: 12.2, range 9.6 to 18.9 and 3 hours p.i.: median SUVpeak: 9.6, range 8.2 to 15.0, ***P = 0.0012). **B** However, bone SUVpeak was significantly increased 3 hours p.i. compared to 1 hours p.i. (1 hours p.i.: median SUVpeak: 3.8, range 3.6 to 4.5 and 3 hours p.i.: median SUVpeak: 5.6, range 4.7 to 6.4; ****P = 0.0007). **C** Retention index similar to myocardial SUVpeak was significantly increased 1 hours p.i. compared to 3 hours p.i. (1 hours p.i.: 4.6, range 2.6 to 5.0 and 3 hours p.i.: 3.0, range 1.9 to 3.7; *****P = 0.04)

Quantitative SPECT/CT imaging at 1 and 3 hours p.i. revealed a significantly increased myocardial SUVpeak in early imaging (1 hours p.i.: median SUVpeak: 12.2, range 9.6 to 18.9 and 3 hours p.i.: median SUVpeak: 9.6, range 8.2 to 15.0, *P* = .0012; Figure 2a), while bone SUVpeak was significantly increased 3 hours p.i (1 hours p.i.: median SUVpeak: 3.8, range 3.6 to 4.5 and 3 hours p.i.: median SUVpeak: 5.6, range 4.7 to 6.4; *P* = .0007; Figure 2b). Additional evaluation of SUVmax and SUVmean showed a significant increase 1 hours p.i. compared to 3 hours p.i. (1 hours p.i. median SUVmax: 13.2, range 10.2 to 19.9 and 3 hours p.i. median SUV max: 10.2, range 9.3 to 15.7; *P* = .0004 and 1 hours p.i. median SUVmean: 7.5, range 5.5 to 11.2 and 3 hours p.i. median SUVmean: 5.8, range 5.0 to 8.8; *P* = .001, Online Resource Figure ESM1 a) and b)).

Furthermore, the threshold based estimated uptake volume 1 hours p.i. compared to 3 hours p.i. was found to be significantly increased (1 hours p.i.:204 ± 87 mL vs 3 hours p.i.: 166 ± 106 mL; *P* = .02) as well as the SUV retention index (1 hours p.i.: 4.6, range 2.6 to 5.0 and 3 hours p.i.: 3.0, range 1.9 to 3.7; *P* = .04; Figure 2c). Finally, we looked at the myocardial SUV peak/bloodpool SUVmean ratio (1 hours p.i.: 9.0 ± 4.3 vs 3 hours p.i.: 80.1 ± 159.6; *P* = .41) without any significant difference, which was in contrast to the significantly higher myocardial SUVpeak/bone SUVpeak ratio in early imaging (1 hours p.i. 3.2 ± 1.4 vs 3 hours p.i. 2.0 ± 1.1; *P* < .0001).

Linear regression analysis showed a significant correlation for myocardial SUVpeak between 1 and 3 hours p.i. (*r*^2^ = 0.76; *P* < .0001), for bone SUVpeak (*r*^2^ = 0.56; *P* = .0013) and for SUV retention index (*r*^2^ = 0.39; *P* = .01). Moreover, a significant linear correlation of the planar imaging based myocardial SUVpeak 3 hours p.i., with the H/CL ratio (1 hours p.i.: *r*^2^ = 0.22; *P* = .03 and 3 hours p.i.: *r*^2^ = 0.22, *P* = .03) as well as with the H/WB ratio (1 hours p.i.: *r*^2^ = 0.48, *P* = .0004 and 3 hours p.i.: *r*^2^ = 0.52, *P* = .0002) was also observed.

## Discussion

Bone scintigraphy has been recently accepted as an imaging tool together with the so called Perugini scoring system for the diagnosis of cATTR.^23^ With the development and introduction of several drugs reducing mortality in patients with cATTR bone scintigraphy may also become an important role in treatment monitoring in the future. Therefore, well-defined and understood quantification methods for this functional imaging are recommended beside a fixed time-point of imaging.

**Figure 3:**
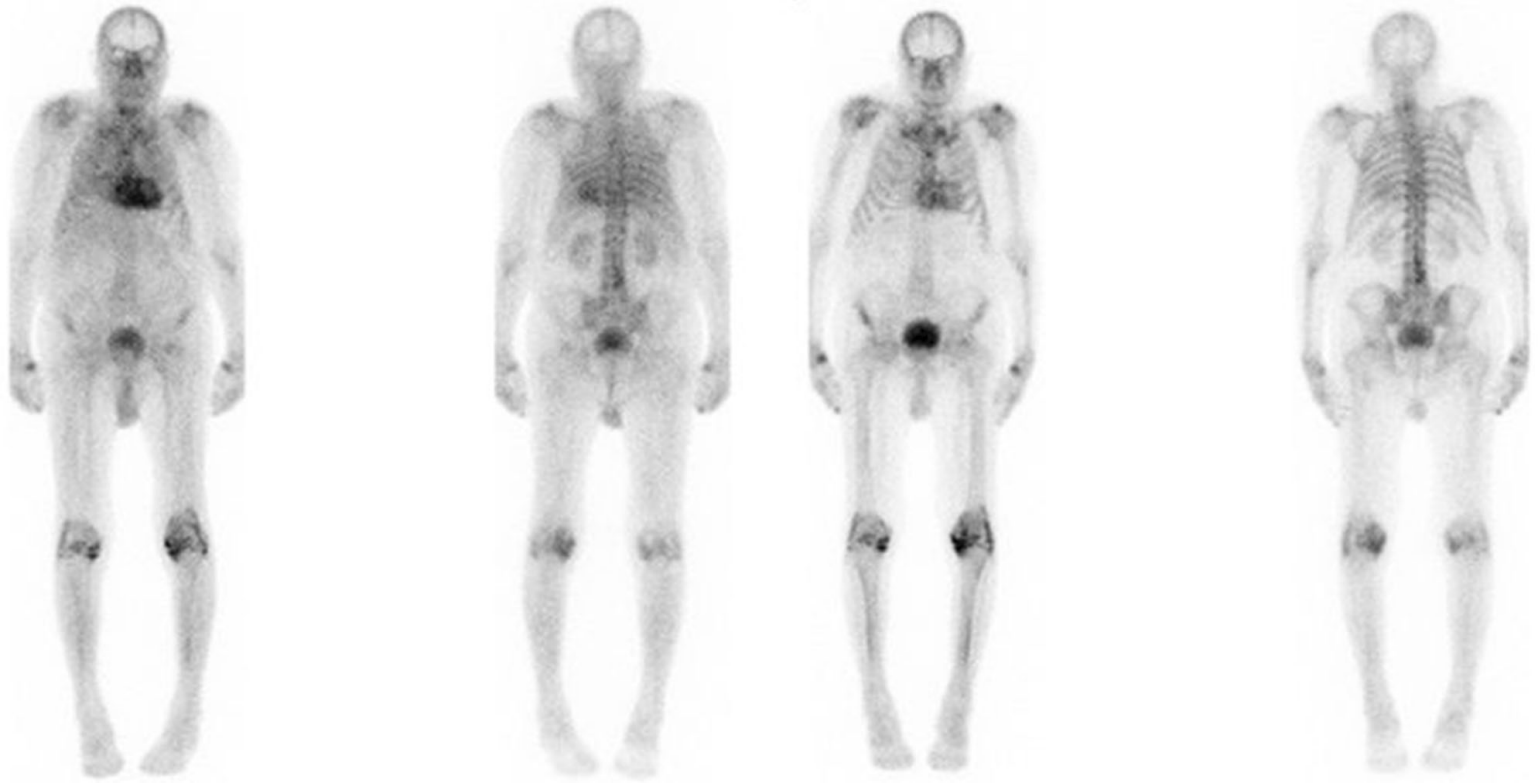
Example images of a patient with increased myocardial uptake 1 hours p.i. compared to 3 hours p.i. Anterior and posterior planar DPD-bone scintigraphy 1 hours and 3 hours p.i. of a patient with strong myocardial and soft tissue (decay corrected geometric mean of the total counts over the heart: 61,922 cts, H/CL ratio: 2.07, H/WB-ratio: 0.053), and mild bone uptake graded as Perugini score 3 1 hours p.i.. (left side). The same patient with moderate myocardial (myocardial SUVpeak 7.7; decay corrected geometric mean of the total counts over the heart: 37,217 cts, H/CL ratio 1.82, H/WB 0.043), reduced soft tissue and mild bone tracer uptake graded as Perugini grade 2 3 hours p.i. (right side)

According to the current Expert Consensus Recommendations for Multimodality Imaging in Cardiac Amyloidosis, radionuclide imaging should be performed early (1 hours p.i.) or late (3 hours p.i.).^23^ However, early imaging in particular can be influenced by blood pool activity. To distinguish between myocardial tracer accumulation and blood pool activity, additional SPECT imaging with or without CT is needed.^23^ Therefore, we investigated different bone scintigraphy analysis tools as possible imaging monitoring tools of cATTR patients. In a first step the Perugini grading 1 and 3 hours p.i. showed significant higher scores in the early phase of imaging with a scoring change in 37,5% of the patients. These cardiac and soft tissue blood pool-based differences are also illustrated in Figure 3 demonstrating a falsely high Perugini grade 3 in early imaging. This observation is also supported by others describing a reduced reader’s confidence for early phase analysis of visual scoring.^24^ Nevertheless, we only revealed a change of Perugini grade 2 and 3, which will not affect the diagnosis of cATTR as Gillmore et al. described with a specificity and positive predictive value for cATTR amyloidosis of 100% combining the findings of grade 2 or 3 Perugini score together with the absence of a monoclonal protein in serum or urine.^25^

Because the exact mechanism of DPD binding to TTR amyloid fibrils is not yet fully elucidated, it is however important to understand the time dependency of the myocardial tracer uptake, especially if bone scintigraphy is used for potential therapy monitoring of patients with cATTR. Hutt et al. described for the first time a cardiac DPD-uptake peak after 1 hours compared to 3 hours p.i. in four patients^16^ we could now confirm in a larger study population observing significantly increased decay corrected counts over the heart 1 hours compared to 3 hours p.i.. Furthermore, investigating semi-quantitative parameters the H/WB ratio was also significantly increased at early imaging after 1 hours compared to 3 hours p.i..

Comparing the H/CL ratios 1 hours p.i to 3 hours p.i. no significant differences were observed in the present study. This in contrast to the results of prior studies like Castano et al.^20^ who described a significant lower H/CL-ratio 3 hours p.i. compared to 1 hours p.i imaging. One explanation for this discrepancy could be the use of Tc99m-PYP instead of DPD with potential different uptake behavior over the imaging time.^17,20,26^ Moreover, we only included and analysed patients with a confirmed diagnosis of ATTR in our study, which is in good agreement with Schatka et al.^24^ who also used DPD as a tracer and revealed only a significant difference in H/CL ratios between 1 and 3 hours only with a negative standard of reference but not in patients with a positive standard of reference corresponding to our patient collective. Moreover, it is possible that due to a strong tracer uptake of amyloid involved lungs (see Figure 3), the H/CL is not as strongly influenced as in patients without ATTR.

Additionally, we also analysed clinical parameters of the study cohort for potential influence on the H/CL rate over time. Patients with HFrEF as well as patients with HFpEF did not show any significant difference between 1 and 3 hours p.i. suggesting that a higher blood pool is not the reason for the non-significant difference in H/CL between 1 and 3 hours p.i. Furthermore, we included patients with wtATTR and hATTR, who may present different tracer distribution behavior^15,27^ with a higher lung uptake in the early imaging phase consecutively influencing the H/CL rations as shown in Figure 3. Examining patients with wtATTR and hATTR separately no significant difference were also observed making it unlikely that a different tissue and compartment composition may affected the H/CL rate at 1 hours p.i. and at 3 hours p.i. Additionally, the significant linear correlation between early and late-phase imaging of both H/WB ratio and H/CL ratio indicates that at a certain time point these values seem to be comparable. This is important to know using one of these values for image analysis, especially in a treatment monitoring setting.

To minimize the influence of the blood pool activity on cardiac tracer uptake, we further performed quantitative SPECT/CT in 15 patients revealing a significantly increased myocardial SUVpeak 1 hours p.i. and a significantly increased bone SUVpeak measured in the vertebra 3 hours p.i, which confirm the results from Hutt et al.,^16^ who observed a peak of the bone uptake 2 to 3 hours p.i. in planar images. The increasing bone uptake may also be the explanation for the decreasing myocardial DPD-uptake due to a competition between both compartments for tracer uptake. This is an important issue to know for longitudinal cardiac amyloidosis imaging, because the increasing bone uptake over time is in contrast to the actual EANM guidelines stating a maximum bone accumulation 1 hours after tracer injection remaining constant up to 72 hours,^28^ which should be reconsidered. To minimize the potential influence of competition between the compartments myocardium, bone and additionally soft tissue we compared the SUV retention index 1 and 3 hours p.i.. The fact, that the retention index was significantly increased in the early phase of imaging, also allows to assume that not only the bone but also the soft tissue influences the uptake behavior of DPD in a larger study population with a more sophisticated protocol than Hutt al. described.^16^ Furthermore the significantly higher myocardial SUVpeak/bone SUVpeak ratio in early imaging indicates that the increasing bone uptake influences the time-dependent uptake difference. In contrast, the myocardial SUVpeak/bloodpool SUVpeak ratio showed no significant difference between 1 and 3 hours p.i. and might be therefore a more stable parameter for the tracer uptake evaluation. However, due to the low blood pool activity at 3 hours p.i. the large standard deviation indicates a high variability of the measured values and therefore the myocardial SUVpeak/ bloodpool SUVpeak ratio is not ideal for the evaluation of the cardiac DPD-uptake. Finally, the 39% threshold based uptake volume 1 hours p.i. was significantly increased compared to 3 hours p.i. also indicating that the metabolic DPD volume is also time-dependent.

## Study limitations

This study suffers from some limitations. Due to the time-consuming imaging procedures in a first-of-all clinical routine setting the number of patients investigated is explained. Nevertheless, almost exclusively patients with strong cardiac tracer uptake were included in the study, as we wanted to investigate the time dependence of the uptake behavior in patients with confirmed cATTR amyloidosis under the aspect of possible therapeutic intervention. Moreover, the low-dose CT component was only performed at 3 hours p.i. for reconstruction of quantitative SPECT/CT images for both imaging time points because of radiation protection reasons. Although this procedure may influence the results of the study, all images were checked for a good overlay of CT and SPECT images.

## New knowledge gained

DPD cardiac uptake is time-dependent as demonstrated by SPECT/CT based SUV quantification. Explained by an increased blood pool activity, but also by a possible stronger binding behavior of DPD to the amyloid deposit at an earlier time point, this observation should be considered for imaging guided therapy monitoring.

## Conclusion

The results of this study revealed that DPD bone imaging in patients with cATTR for grading of the disease burden is affected by time-dependent cardiac, bone and soft-tissue tracer uptake kinetics using different imaging based analysis tools in a well-defined study cohort. Beside visual Perugini score changes in ~ 38% of cATTR patients also quantitative parameter are influenced. The observed significantly higher myocardial SUVpeak and the SUV retention index 1 hours p.i. compared to 3 hours p.i, seem to be influenced not only by an elevated blood pool tracer activity but also by the binding behavior of DPD itself. Therefore, only a standardized DPD imaging protocol at a fixed time such as 3 hours p.i. should be considered with not only planar, but also SPECT/CT based quantification especially in the setting of serial treatment-based monitoring in patients with cATTR.

## Supplementary Information

Below is the link to the electronic supplementary material.

Supplementary file1 **Fig. ESM1: Time-dependent DPD tracer-uptake kinetics using quantitative SPECT analysis (SUVmax and SUVmean)** a) Myocardial SUVmax as well as b) SUVmean were significantly increased 1h p.i. compared to 3h p.i. (SUVmax 1h p.i: 13.2, range: 10.2-19.9 and 3h p.i.: 10.2, range: 9.3-15.7, *p=0.0004; SUVmean 1h p.i: 7.5, range: 5.5-11.2 and 3h p.i.: 5.8, range: 5.0-8.8, **p=0.0006). (TIF 317 kb)

Supplementary file2 (PPTX 702 kb)

## Abbreviations

AL: Light-chain amyloidosis
wtATTR: Wild-type transthyretin amyloidosis
hATTR: Hereditary transthyretin amyloidosis
SUV: Standard uptake values
HMDP: Hydroxymethylene diphosphonate
DPD: 3,3-Diphosphono-1,2-propanodicarboxylic acid
H/CL: Heart to contralateral
H/CL: Heart to contralateral
ROI: Region of interest
cATTR: Cardiac ATTR
