# Supplementary figures and images for: Cardiac DPD-uptake time dependency in ATTR patients verified by quantitative SPECT/CT and semiquantitative planar parameters
Source: J Nucl Cardiol. 2022 Dec 13;30(4):1363–71. doi: 10.1007/s12350-022-03149-4 (PMC10371940; doi:10.1007/s12350-022-03149-4)

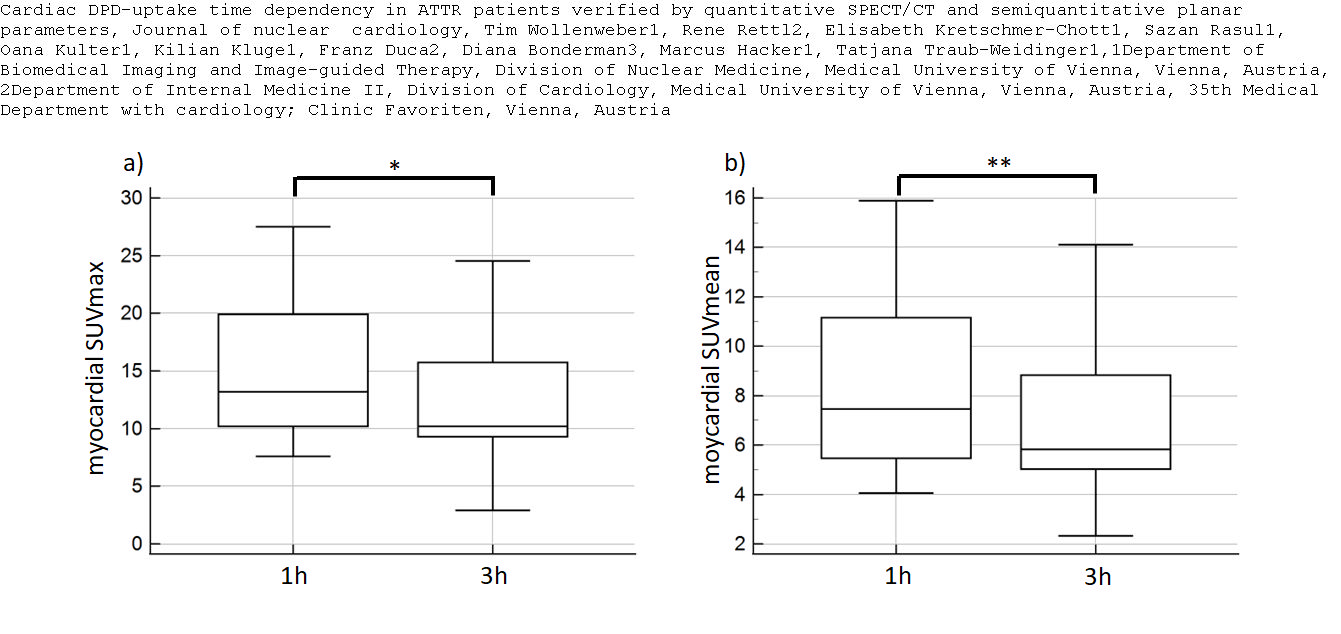

Supplement: Supplementary file 1 — Supplementary file1 Fig. ESM1: Time-dependent DPD tracer-uptake kinetics using quantitative SPECT analysis (SUVmax and SUVmean) a) Myocardial SUVmax as well as b) SUVmean were significantly increased 1h p.i. compared to 3h p.i. (SUVmax 1h p.i: 13.2, range: 10.2-19.9 and 3h p.i.: 10.2, range: 9.3-15.7, *p=0.0004; SUVmean 1h p.i: 7.5, range: 5.5-11.2 and 3h p.i.: 5.8, range: 5.0-8.8, **p=0.0006). (TIF 317 kb) [file 12350_2022_3149_MOESM1_ESM.tif]
