# Supplementary material for: Cardiac DPD-uptake time dependency in ATTR patients verified by quantitative SPECT/CT and semiquantitative planar parameters
Source: J Nucl Cardiol. 2022 Dec 13;30(4):1363–71. doi: 10.1007/s12350-022-03149-4 (PMC10371940; doi:10.1007/s12350-022-03149-4)
Supplement: Supplementary file 2 — Supplementary file2 (PPTX 702 kb) [file 12350_2022_3149_MOESM2_ESM.pptx]

## Slide 1
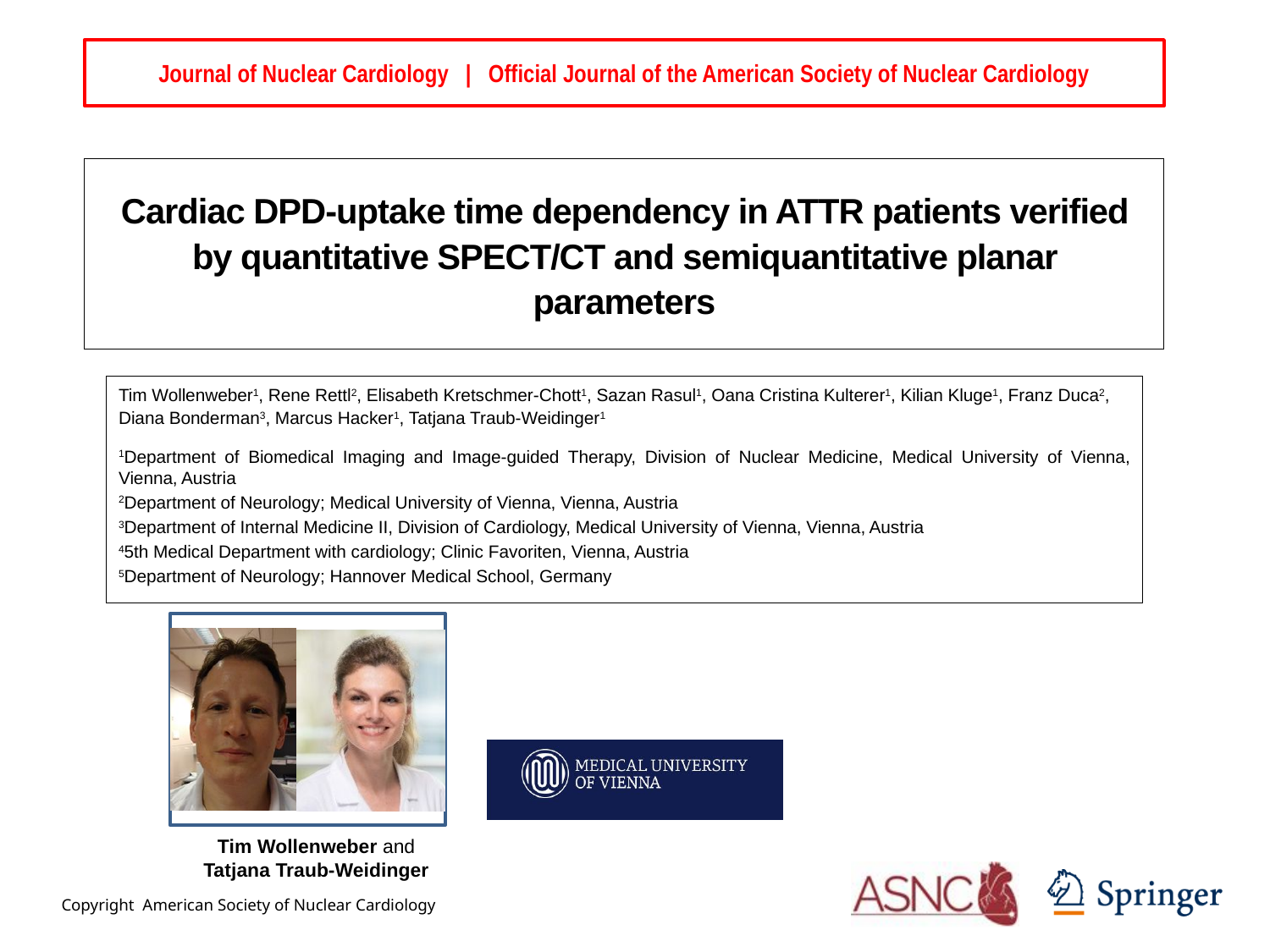

Journal of Nuclear Cardiology | Official Journal of the American Society of Nuclear Cardiology
# Cardiac DPD-uptake time dependency in ATTR patients verified by quantitative SPECT/CT and semiquantitative planar parameters
Tim Wollenweber1, Rene Rettl2, Elisabeth Kretschmer-Chott1, Sazan Rasul1, Oana Cristina Kulterer1, Kilian Kluge1, Franz Duca2, Diana Bonderman3, Marcus Hacker1, Tatjana Traub-Weidinger1
1Department of Biomedical Imaging and Image-guided Therapy, Division of Nuclear Medicine, Medical University of Vienna, Vienna, Austria
2Department of Neurology; Medical University of Vienna, Vienna, Austria
3Department of Internal Medicine II, Division of Cardiology, Medical University of Vienna, Vienna, Austria
45th Medical Department with cardiology; Clinic Favoriten, Vienna, Austria
5Department of Neurology; Hannover Medical School, Germany
Head shot of author
required
Tim Wollenweber and Tatjana Traub-Weidinger
Copyright American Society of Nuclear Cardiology

## Slide 2
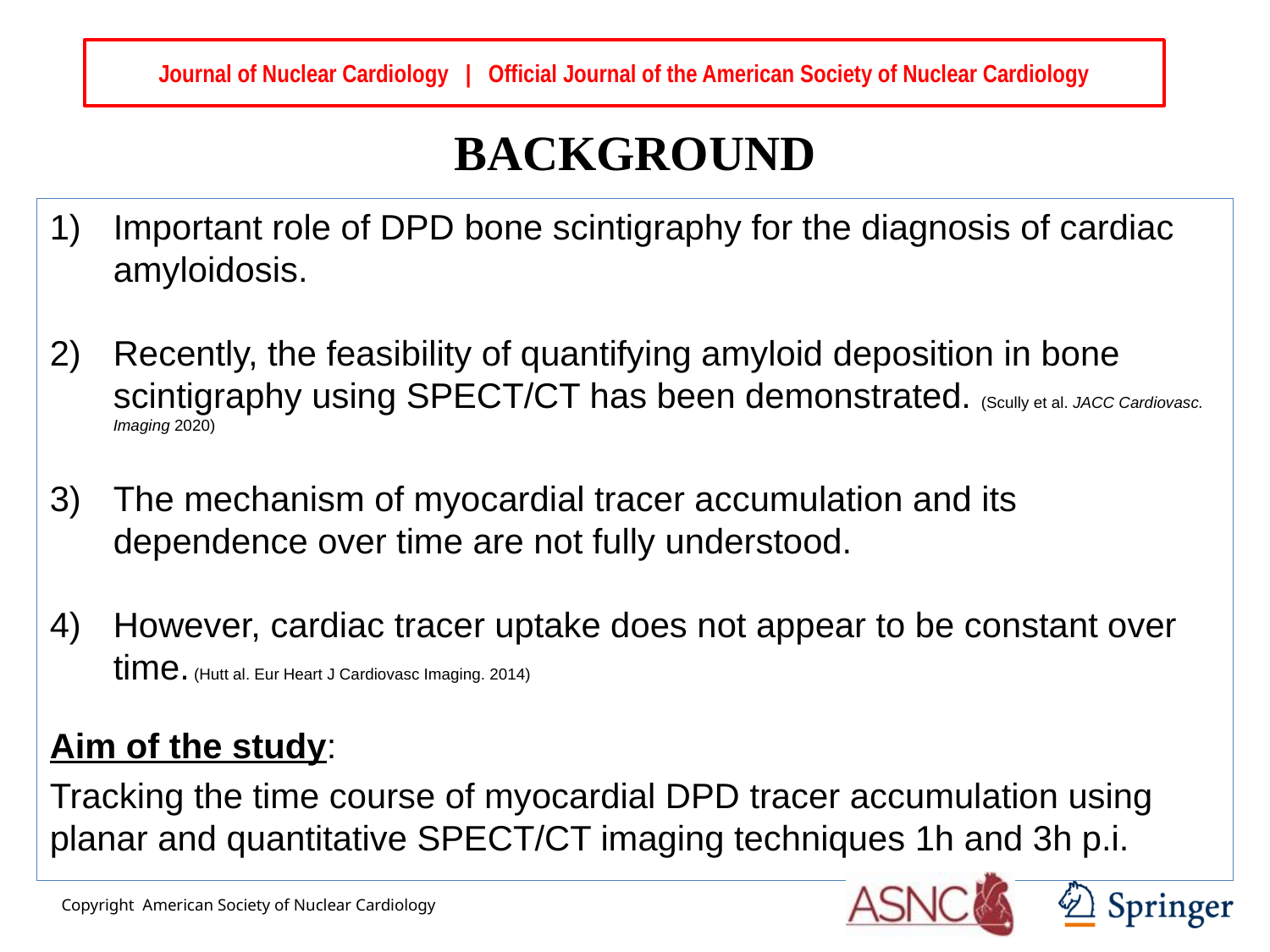

Journal of Nuclear Cardiology | Official Journal of the American Society of Nuclear Cardiology
# BACKGROUND
Important role of DPD bone scintigraphy for the diagnosis of cardiac amyloidosis.
Recently, the feasibility of quantifying amyloid deposition in bone scintigraphy using SPECT/CT has been demonstrated. (Scully et al. JACC Cardiovasc. Imaging 2020)
The mechanism of myocardial tracer accumulation and its dependence over time are not fully understood.
However, cardiac tracer uptake does not appear to be constant over time. (Hutt al. Eur Heart J Cardiovasc Imaging. 2014)
Aim of the study:
Tracking the time course of myocardial DPD tracer accumulation using planar and quantitative SPECT/CT imaging techniques 1h and 3h p.i.
Copyright American Society of Nuclear Cardiology

## Slide 3
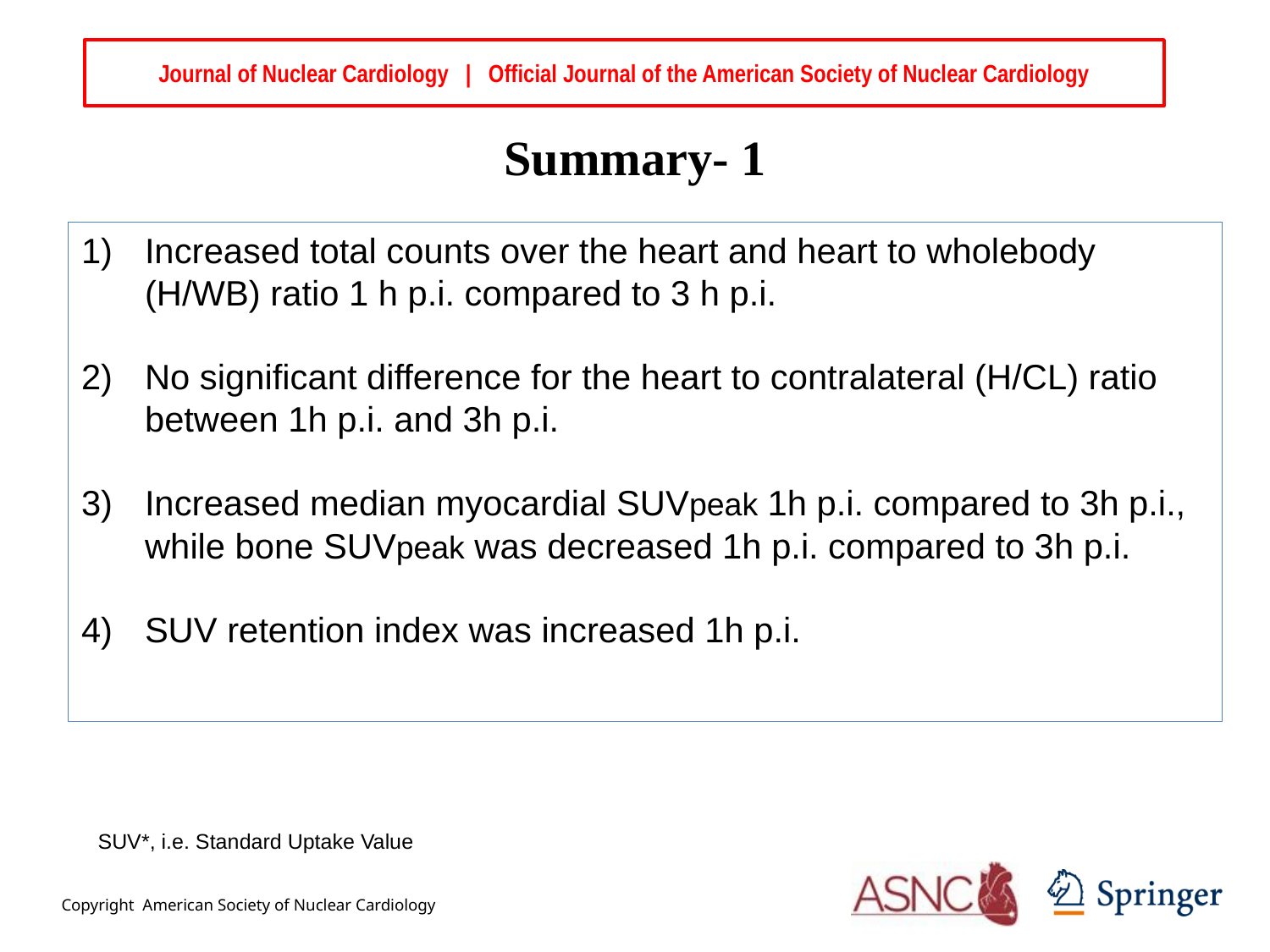

Journal of Nuclear Cardiology | Official Journal of the American Society of Nuclear Cardiology
# Summary- 1
Increased total counts over the heart and heart to wholebody (H/WB) ratio 1 h p.i. compared to 3 h p.i.
No significant difference for the heart to contralateral (H/CL) ratio between 1h p.i. and 3h p.i.
Increased median myocardial SUVpeak 1h p.i. compared to 3h p.i., while bone SUVpeak was decreased 1h p.i. compared to 3h p.i.
SUV retention index was increased 1h p.i.
SUV*, i.e. Standard Uptake Value
Copyright American Society of Nuclear Cardiology

## Slide 4
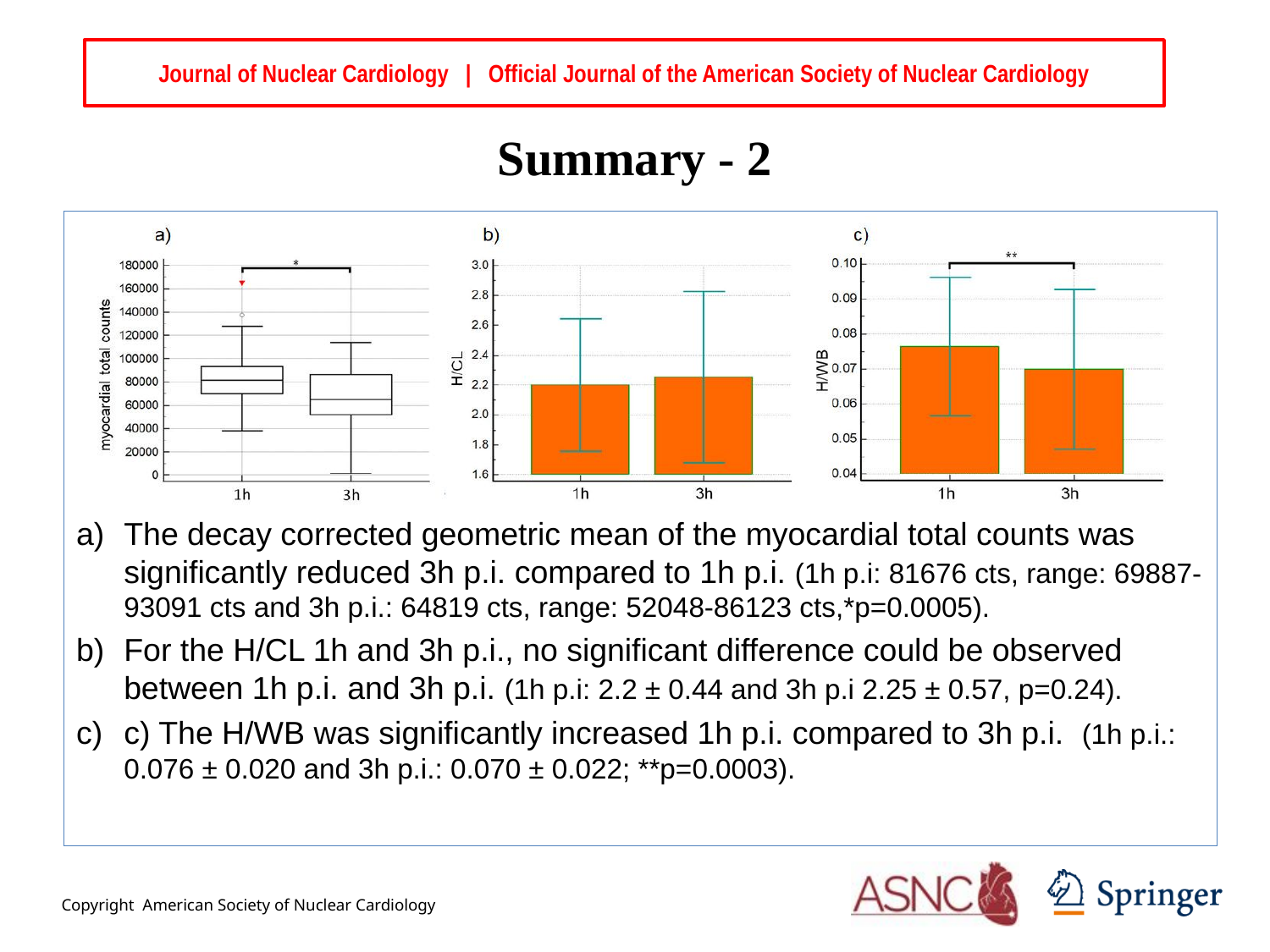

Journal of Nuclear Cardiology | Official Journal of the American Society of Nuclear Cardiology
# Summary - 2
The decay corrected geometric mean of the myocardial total counts was significantly reduced 3h p.i. compared to 1h p.i. (1h p.i: 81676 cts, range: 69887-93091 cts and 3h p.i.: 64819 cts, range: 52048-86123 cts,*p=0.0005).
For the H/CL 1h and 3h p.i., no significant difference could be observed between 1h p.i. and 3h p.i. (1h p.i: 2.2 ± 0.44 and 3h p.i 2.25 ± 0.57, p=0.24).
c) The H/WB was significantly increased 1h p.i. compared to 3h p.i. (1h p.i.: 0.076 ± 0.020 and 3h p.i.: 0.070 ± 0.022; **p=0.0003).
Copyright American Society of Nuclear Cardiology

## Slide 5
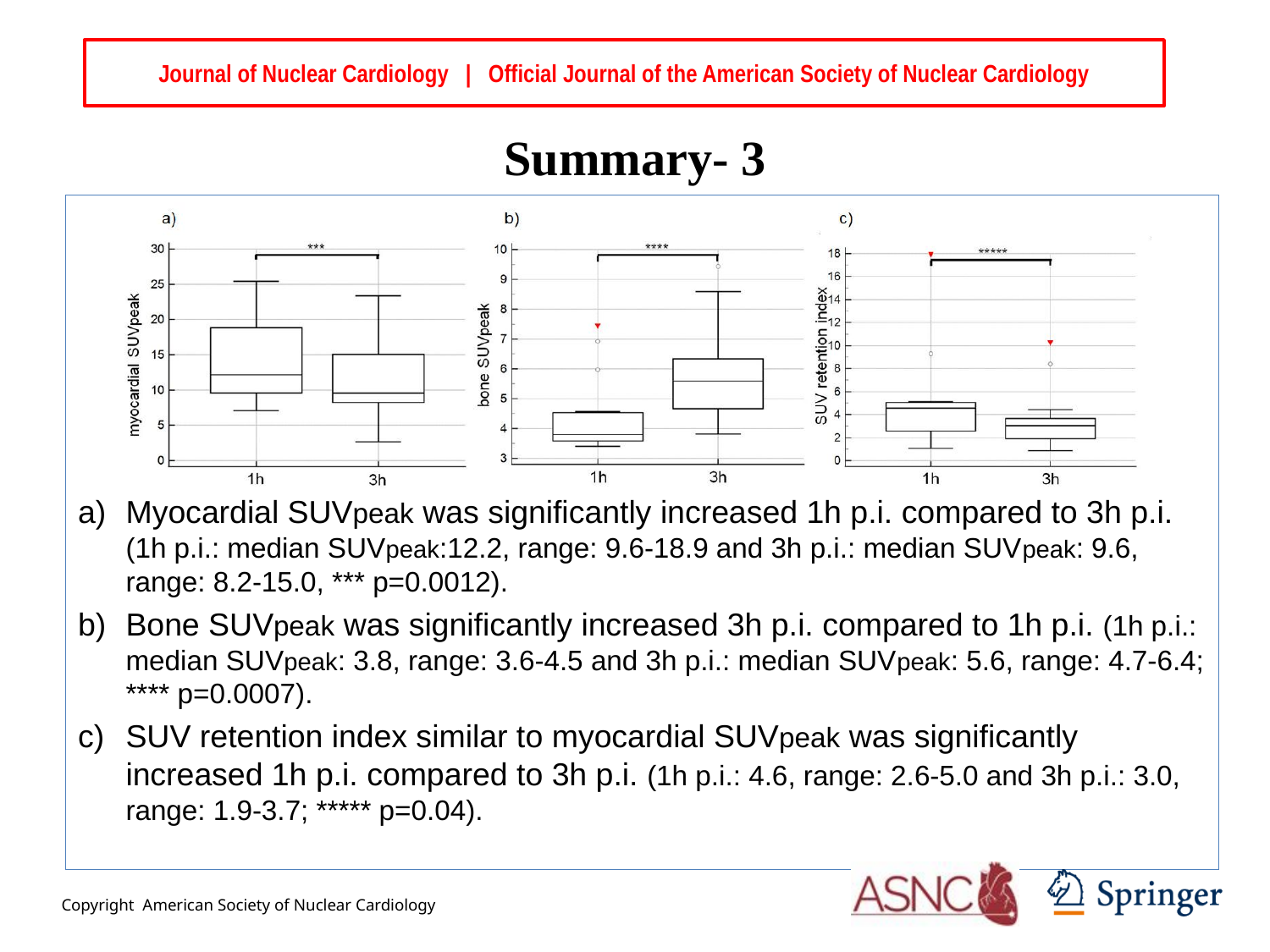

Journal of Nuclear Cardiology | Official Journal of the American Society of Nuclear Cardiology
# Summary- 3
Myocardial SUVpeak was significantly increased 1h p.i. compared to 3h p.i. (1h p.i.: median SUVpeak:12.2, range: 9.6-18.9 and 3h p.i.: median SUVpeak: 9.6, range: 8.2-15.0, *** p=0.0012).
Bone SUVpeak was significantly increased 3h p.i. compared to 1h p.i. (1h p.i.: median SUVpeak: 3.8, range: 3.6-4.5 and 3h p.i.: median SUVpeak: 5.6, range: 4.7-6.4; **** p=0.0007).
SUV retention index similar to myocardial SUVpeak was significantly increased 1h p.i. compared to 3h p.i. (1h p.i.: 4.6, range: 2.6-5.0 and 3h p.i.: 3.0, range: 1.9-3.7; ***** p=0.04).
Copyright American Society of Nuclear Cardiology

## Slide 6
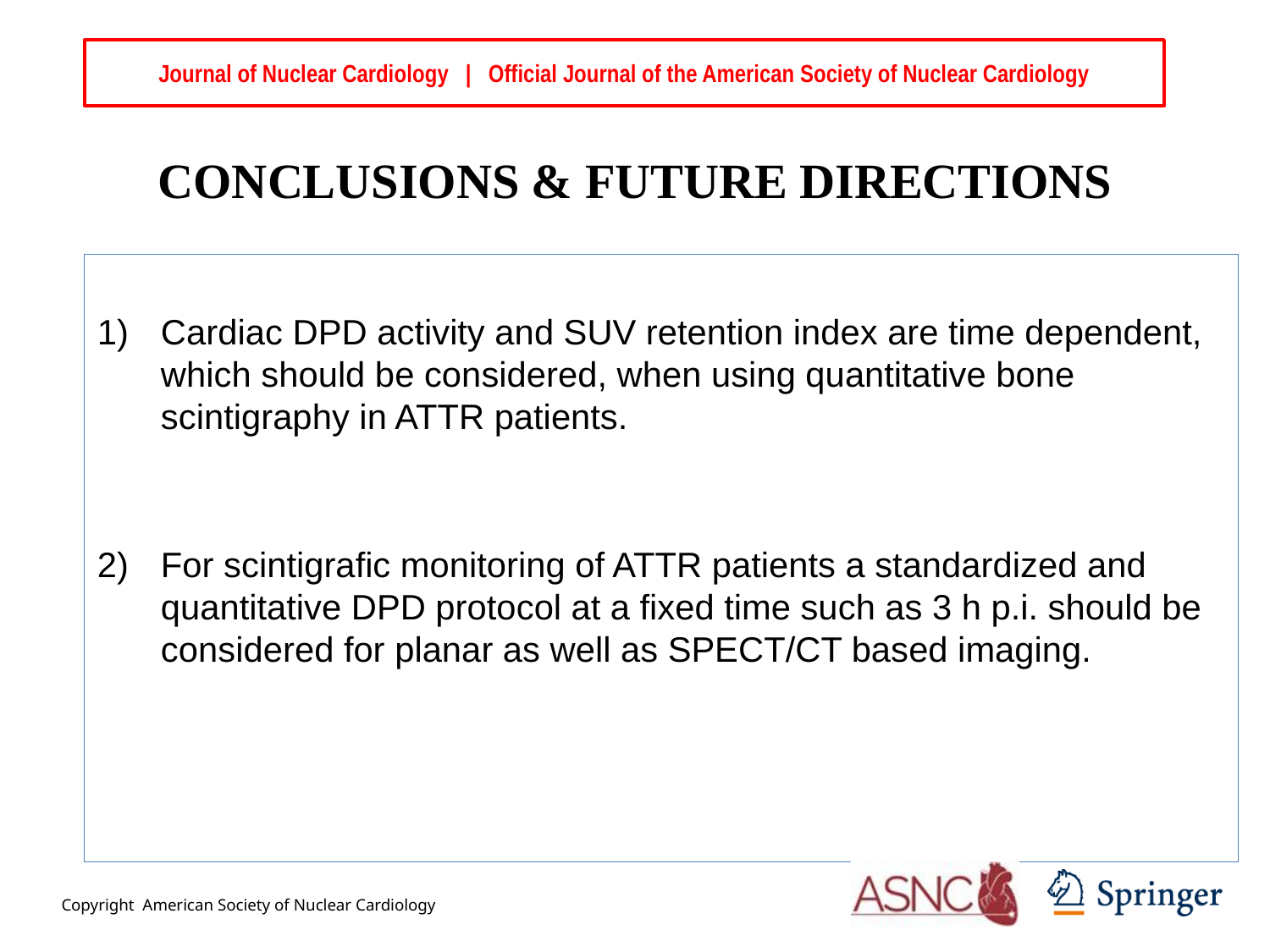

Journal of Nuclear Cardiology | Official Journal of the American Society of Nuclear Cardiology
# CONCLUSIONS & FUTURE DIRECTIONS
Cardiac DPD activity and SUV retention index are time dependent, which should be considered, when using quantitative bone scintigraphy in ATTR patients.
For scintigrafic monitoring of ATTR patients a standardized and quantitative DPD protocol at a fixed time such as 3 h p.i. should be considered for planar as well as SPECT/CT based imaging.
Copyright American Society of Nuclear Cardiology
